# Feature analysis for classification of trace fluorescent labeled protein crystallization images

**DOI:** 10.1186/s13040-017-0133-9

**Published:** 2017-04-27

**Authors:** Madhav Sigdel, Imren Dinc, Madhu S. Sigdel, Semih Dinc, Marc L. Pusey, Ramazan S. Aygun

**Affiliations:** 1 Computer Science Department, University of Alabama in Huntsville, Huntsville, 35899 Alabama USA; 2 Computer Science Department, Troy University, Troy, 36082 Alabama USA; 3 iXpressGenes, Inc., 601 Genome Way, Huntsville, 35806 Alabama USA

**Keywords:** Protein crystallization, Image classification, Feature analysis, Trace-fluorescent labeling

## Abstract

**Background:**

Large number of features are extracted from protein crystallization trial images to improve the accuracy of classifiers for predicting the presence of crystals or phases of the crystallization process. The excessive number of features and computationally intensive image processing methods to extract these features make utilization of automated classification tools on stand-alone computing systems inconvenient due to the required time to complete the classification tasks. Combinations of image feature sets, feature reduction and classification techniques for crystallization images benefiting from trace fluorescence labeling are investigated.

**Results:**

Features are categorized into intensity, graph, histogram, texture, shape adaptive, and region features (using binarized images generated by Otsu’s, green percentile, and morphological thresholding). The effects of normalization, feature reduction with principle components analysis (PCA), and feature selection using random forest classifier are also analyzed. The time required to extract feature categories is computed and an estimated time of extraction is provided for feature category combinations. We have conducted around 8624 experiments (different combinations of feature categories, binarization methods, feature reduction/selection, normalization, and crystal categories). The best experimental results are obtained using combinations of intensity features, region features using Otsu’s thresholding, region features using green percentile *G*_90_ thresholding, region features using green percentile *G*_99_ thresholding, graph features, and histogram features. Using this feature set combination, 96% accuracy (without misclassifying crystals as non-crystals) was achieved for the first level of classification to determine presence of crystals. Since missing a crystal is not desired, our algorithm is adjusted to achieve a high sensitivity rate. In the second level classification, 74.2% accuracy for (5-class) crystal sub-category classification. Best classification rates were achieved using random forest classifier.

**Contributions:**

The feature extraction and classification could be completed in about 2 s per image on a stand-alone computing system, which is suitable for real time analysis. These results enable research groups to select features according to their hardware setups for real-time analysis.

## Introduction

Protein crystallization is a highly empirical process that depends on numerous factors such as pH and temperature of the environment, protein concentration, the type of precipitant, ionic strength of the solution, gravity, the crystallization methods, etc. [1] A combination of all these factors suitable for the protein being crystallized is critical for the formation of crystals, and the prediction of these parameters is quite challenging since there is no prior information about the protein solubility [2, 3]. Therefore, thousands of experimental trials may be required for successful crystallization. Today, high-throughput robotic systems are routinely used to increase the chance of successfully obtaining crystals. Because of the high throughput crystallization trials, manual review of crystallization trials becomes practically discouraging in terms of time and resources. Therefore, automated image scoring systems have been developed to collect and classify the crystallization trial images. The fundamental aim is to discard the unsuccessful trials, identify the successful trials, and possibly identify those trials which could be optimized.

### Challenges of protein crystallization classification

Imaging techniques are used to capture the state change or the possibility of forming crystals [4]. Building a reliable system to classify and analyze the crystallization trial can be very helpful to the crystallographers by reducing the number of tedious manual reviews of unsuccessful outcomes or providing the phase of the crystallization process. Such a system requires extracting features from images. After these features are used to train a classifier, the classifier model is used to classify new trial images. However, building a classifier model with high accuracy is challenging due to following reasons.

1. **Many Phases of Crystallization Process.** The instruction sheets with crystallization screens from Hampton Research describe 9 possible protein crystallization trial outcomes or phases^1^ [5] (Clear drop, Phase separation, Granular precipitate, Microcrystals, Posettes/spherulites, Needles, 2D Plates, Small 3D crystals, Large 3D crystals). Figure 1 shows sample protein crystallization trial images obtained using trace fluorescence labeling [6] where each image corresponds to a specific phase of crystallization. In analysis of the screening images, it is important to predict/detect the current phase of the experiment. Phases that yield crystalline outcomes or likely-leads are more valuable than other categories. Misclassification of the images in a higher category (e.g., crystal category) into a lower category (e.g., non-crystal category) is a serious problem as it results in a lead condition being missed. The misclassification of a lower category result to a higher is not as serious, and can be considered as a cost of capturing all possible leads.

**Fig. 1:**
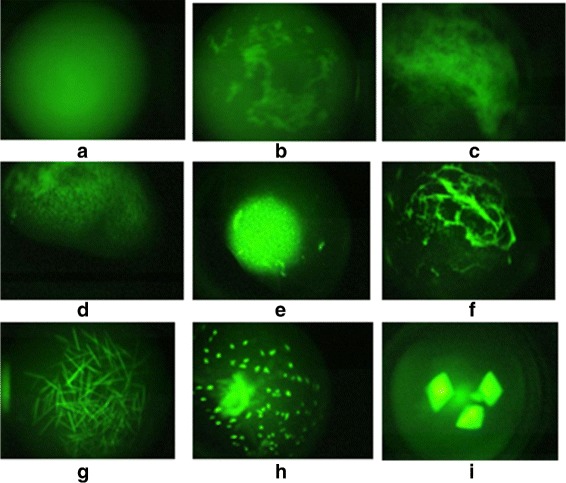
Sample protein crystallization trial images **a**-**c**) non-crystals, **d**-**f**) likely-leads, and **g**-**i**) crystals. Reprinted with permission from [28]. Copyright 2013 American Chemical Society
2. **Unbalanced Distribution of Data.** The distribution of data in different categories (or phases) is unbalanced. Frequency of higher (crystalline) categories are less than the frequency of lower categories. The classification models can be affected adversely by the unbalanced distribution. They may classify in favor of more frequent but less important categories.
3. **Complexity of Image Analysis.** Non-uniform shapes and varying orientation of crystals impose complexity in image analysis. Intra-class diversity of a single crystal sub-category is significantly high. It is difficult to build a classifier with high accuracy that can model all variations.
4. **Multiple Types of Crystals in a Single Image.** A single image can consist of objects (crystals) in different morphologies, such as dendrites and 3D crystals. In such cases, the expected class for the image would be the class corresponding to the highest class among all crystal objects.
5. **Low and Varying Image Quality.** Since crystals are floating in a 3D well, not all crystals may be captured in focus. To observe the phases of crystallization, images are captured a number of times during the process. The lighting conditions may vary each time the images are collected. Varying illumination and focusing affect the pre-processing of images and features used for classification.
6. **Ambiguity in Labeling Trial Images.** Protein crystallization is an evolving process. In some scenarios, there is a semantic transition between categories, meaning the images cannot be clearly assigned to one category. Similarly, ambiguities and subjectivity of the viewer or an expert can affect the labeling process or expert scoring.

### Related work

In general, protein crystallization trial image analysis work is compared with respect to the accuracy of classification. The accuracy depends on the number of categories, features, and the ability of classifiers to model the data. Moreover, the hardware resources, training time and real-time analysis of new images are important factors that affect the usability of these methods. Table 1 provides the summary of related work with respect to different factors.

**Table 1:** Summary of related work

Research paper	Image categories	Feature extraction	Classification method	Classification accuracy
Zuk and Ward (1991) [7]	NA	Edge features	Detection of lines using Hough transform and line tracking	Not provided
Walker et al. (2007) [22]	7	Radial and angular descriptors from Fourier Transform	Learning vector quantization	14 - 97% for different categories
Xu et al. (2006) [23]	2	Features from multiscale Laplacian pyramid filters	Neural network	95% accuracy
Wilson (2002) [24]	3	Intensity and geometric features	Naive Bayes	Recall 86% for crystals, 77% for unfavourable objects
Hung et al. (2014) [26]	3	Shape context, Gabor filters and Fourier transforms	Cascade classifier on naive Bayes and random forest	74% accuracy
Spraggon et al. (2002) [17]	6	Geometric and texture features	Self-organizing neural networks	47 to 82% for different categories
Cumba et al. (2003) [8]	2	Radon transform line features and texture features	Linear discriminant analysis	85% accuracy with roc 0.84
Saitoh et al. (2004) [20]	5	Geometric and texture features	Linear discriminant analysis	80 - 98% for different categories
Bern et al. (2004) [15]	5	Gradient and geometric features	Decision tree with hand crafted thresholds	12% FN and 14% FP
Cumba et al. (2005) [9]	2	Texture features, line measures and energy measures	Association rule mining	85% accuracy with ROC 0.87
Zhu et al. (2004) [10]	2	Geometric and texture features	Decision tree with boosting	14.6% FP and 9.6% FN
Berry et al. (2006) [11]	2	NA	Learning vector quantization, self organizing maps and bayesian algorithm	NA
Pan et al. (2006) [12]	2	Intensity stats, texture features, Gabor wavelet decomposition	Support vector machine	2.94% FN and 37.68% FP
Yang et al. (2006) [14]	3	Hough transform, DFT, GLCM features	Hand tuned thresholds	85% accuracy
Saitoh et al. (2006) [16]	5	Texture features, differential image features	Decision tree and SVM	90% for 3-class problem
Po and Laine (2008) [13]	2	Multiscale Laplacian pyramid filters and histogram analysis	Genetic algorithm and neural network	Accuracy: 93.5% with 88% TP and 99% TN
Liu et al. (2008) [21]	Crystal likelihood	Features from Gabor filters, integral histograms, and gradient images	Decision tree with boosting	ROC 0.92
Cumba et al. (2010) [18]	3 and 6	Basic stats, energy, Euler numbers, Radon-Laplacian, Sobel-edge, GLCM	Multiple random forest with bagging and feature subsampling	Recall 80% crystals, 89% precipitate, 98% clear drops
Sigdel et al. (2013) [28]	3	Intensity and blob features	Multilayer perception neural network	1.2% crystal misses with 88% accuracy
Sigdel et al. (2014) [25]	3	Intensity and blob features	Semi-supervised	75% - 85% overall accuracy
Dinc et al. (2014) [27]	3 and 2	Intensity and blob features	5 classifiers, feature reduction using PCA	96% on non-crystals, 95% on likely-leads
Yann et al. (2016) [19]	10	Deep learnining on grayscale image	Deep CNN with 13 layers	90.8% accuracy

*The Number of Categories.* A significant amount of previous work (for example, Zuk and Ward (1991) [7], Cumba et al. (2003) [8], Cumba et al. (2005) [9], Zhu et al. (2006) [10], Berry et al. (2006) [11], Pan et al. (2006) [12], Po and Laine (2008) [13]) classified crystallization trials into non-crystal or crystal categories. Yang et al. (2006) [14] classified the trials into three categories (clear, precipitate, and crystal). Bern et al. (2004) [15] classified the images into five categories (empty, clear, precipitate, microcrystal hit, and crystal). Likewise, Saitoh et al. (2006) [16] classified into five categories (clear drop, creamy precipitate, granulated precipitate, amorphous state precipitate, and crystal). Spraggon et al. (2002) [17] proposed classification of the crystallization images into six categories (experimental mistake, clear drop, homogeneous precipitant, inhomogeneous precipitant, micro-crystals, and crystals). Cumba et al. (2010) [18] developed a system that classifies the images into three or six categories (phase separation, precipitate, skin effect, crystal, junk, and unsure). Yann et al. (2016) [19] classified into 10 categories (clear, precipitate, crystal, phase, precipitate and crystal, precipitate and skin, phase and crystal, phase and precipitate, skin, and junk). It should be noted that there is no standard for categorizing the images, and different research studies proposed different categories in their own way. Hampton’s scheme specifies 9 possible outcomes of crystallization trials. We intend to classify the crystallization trials according to Hampton’s scale.

*Features for Classification.* For feature extraction, a variety of image processing techniques have been proposed. Zuk and Ward (1991) [7] used the Hough transform to identify straight edges of crystals. Bern et al. (2004) [15] extract gradient and geometry-related features from the selected drop. Pan et al. (2006) [12] used intensity statistics, blob texture features, and results from Gabor wavelet decomposition to obtain the image features. Research studies by Cumba et al. (2003) [8], Saitoh et al. (2004) [20], Spraggon et al. (2002) [17], and Zhu et al. (2004) [10] used a combination of geometric and texture features as the input to their classifier. Saitoh et al. (2006) [16] used global texture features as well as features from local parts in the image and features from differential images. Yang et al. (2006) [14] derived the features from gray-level co-occurrence matrix, Hough transform and discrete fourier transform (DFT). Liu et al. (2008) [21] extracted features from Gabor filters, integral histograms, and gradient images to obtain 466-dimensional feature vector. Po and Laine (2008) [13] applied multiscale Laplacian pyramid filters and histogram analysis techniques for feature extraction. Similarly, other extracted image features included Hough transform features [13], Discrete Fourier Transform features [22], features from multiscale Laplacian pyramid filters [23], histogram analysis features [9], Sobel-edge features [24], etc. Cumba et al. (2010) [18] presented the most sophisticated feature extraction techniques for the classification of crystallization trial images. Features such as basic statistics, energy, Euler numbers, Radon-Laplacian features, Sobel-edge features, microcrystal features, and gray-level co-occurrence matrix features were extracted to obtain a 14,908 dimensional feature vector. They utilized a web-based distributed system and extracted as many features as possible hoping that the huge set of features could improve the accuracy of the classification [18].

*Time Analysis of Classification.* Because of the high-throughput rate of image collection, the speed of processing an image becomes an important factor. The system by Pan et al. (2006) [12] required 30s per image for feature extraction. Po and Laine mentioned that it took 12.5s per image for the feature extraction in their system [13]. Because of high computational requirement, they considered implementation of their approach on the Google computing grid. Feature extraction described by Cumba et al. (2010) [18] is the most sophisticated, which could take 5 h per image on a normal system. To speed up the process, they executed the feature extraction using a web-based distributed computing system. Yann et al. (2016) [19] utilized deep convolutional neural network (CNN) where training took 1.5 days for 150,000 weights and around 300 passes and classification takes 86 ms for 128x128 image on their GPU-based system.

*Classifiers for Protein Crystallization*. To obtain the decision model for classification, various classification technique have been used. Zhu, et al. (2004) [10] and Liu et al. (2008) [21] applied a decision tree with boosting. Bern et al. (2004) [15] used a decision tree classifier with hand-crafted thresholds. Pan et al. (2006) [12] applied a support vector machines (SVM) learning algorithm. Saitoh et al. (2006) [16] applied a combination of decision tree and SVM classifiers. Spraggon et al. (2002) [17] applied self-organizing neural networks. Po et al. (2008) [13] combined genetic algorithms and neural networks to obtain a decision model. Berry et al. (2006) [11] determined scores for each object within a drop using self-organizing maps, learning vector quantization, and Bayesian algorithms. The overall score for the drop was calculated by aggregating the classification scores of individual objects. Cumba et al. (2003) [8] and Saitoh et al. (2004) [20] applied linear discriminant analysis. Yang et al. (2006) [14] applied hand-tuned rules based classification followed by linear discriminant analysis. Cumba et al. (2005) [9] used association rule mining, while Cumba et al. (2010) [18] used multiple random forest classifiers generated via bagging and feature subsampling. In [25], classification performance using semi-supervised approaches was investigated. The recent study by Hung et al. (2014) [26] proposed protein crystallization image classification using elastic net. In our previous work [27], we evaluated the classification performance using 5 different classifiers, and feature reduction using principal components analysis (PCA) and normalization methods for the non-crystal and likely-lead datasets. Yann et al. (2016) [19] utilized deep convolutional neural networks (CNN) with 13 layers: 0) 128x128 image, 1) contrast normalization, 2) horizontal mirroring, 3) transformation, 4) convolution (5x5 filter), 5) max pooling (2x2 filter), 6) convolution (5x5 filter), 7) max pooling (2x2 filter), 8) convolution (5x5 filter), 9) max pooling (2x2 filter), 10) convolution (3x3 filter), 11) 2048 node fully connected layer, 12) 2048 fully connected layer for rectified linear activation, and 13) output layer using softmax.

*Accuracy of Classification*. With regard to the correctness of a classification, the best reported accuracy for the binary classification (i.e., classification into two categories) is 96.56*%* (83.6% true positive rate and 99.4% true negative rate) using deep CNN [19]. Despite high accuracy rate, around 16% of crystals are missed. Using genetic algorithms and neural networks [13], an accuracy of 93.5*%* average true performance (88% true positive and 99% true negative rates) is achieved for binary classification. Saitoh et al. achieved accuracy in the range of 80−98*%* for different image categories [20]. Likewise, the automated system by Cumba et al. (2010) [18] detected 80% of crystal-bearing images, 89% of precipitate images, and 98% of clear drops accurately. The accuracy also depends on the number of categories. As the number of categories increases, the accuracy goes down since there are more misclassifications possible. For 10-way classification using deep CNN, Yann et al. [19] achieved 91% accuracy with around 76.85*%* true positive rate for crystals and 8% of crystals categorized into classes not related to crystals. While overall accuracy is important, true positive rate (recall or sensitivity) for crystals may carry more value. As crystallographers would like to trust these automated classification systems, it is not desirable to see successful crystalline cases are missed by these systems.

In this study, we will look into whether it is possible to achieve high accuracy with a small set of feature set using a proper classifier considering as many as 10 categories for real-time analysis. We provide an exhaustive set of experiments using all feature combinations and representative classifiers to achieve real-time analysis.

### Feature analysis for building real-time classifiers

The task of building classifier models with high accuracy in the presence of aforementioned issues is challenging. To improve the classification performance, there has been a trend to increase the number of image features and size of datasets. Since it is not known which features may be helpful, all possible features that can be extracted are used to train classifiers hoping that irrelevant features are automatically eliminated or given low weights by the classifiers. For example, Cumba et al. (2010) [18] extracted 14,908 dimensional feature vector per image for classifying protein crystallization images. Overall, the image processing and feature extraction have been computationally expensive for huge number of features making it unfeasible for real time processing. Such systems employ high-performance, grid, distributed or cloud computing systems for manipulating large feature sets. Acquisition of high-end, high-performance and expensive computing systems becomes a barrier for small research labs with limited resources and budget to develop and experiment new promising ideas in a timely manner.

Since extracting numerous features puts a significant computational burden on a typical stand-alone computing system, experts may need to wait for hours before seeing the classification results. Reduction of features is inevitable for building real time classifiers. A wide number of techniques used white light imaging for extracting features.The feature extraction and image processing is cumbersome for white light images. In our experiments, we use an in-house developed Crystal X2 [28] system, and analyze captured images of trace fluorescence labeled protein [6]. The crystal regions have high intensity in images where trace fluorescence labeling is used. The high contrast between the background and the crystals alleviates the image processing and feature extraction. Hence, the number of features can be reduced significantly. Another reason for feature reduction is that the use of irrelevant features may deteriorate the performance of some classifiers. Therefore, it is very important to determine the minimal set of image features that can be used to obtain a reliable classification performance.

Herein, we investigate the image features, feature reduction techniques and classification techniques for the images captured using trace fluorescence labeling. We experiment with a number of feature set combinations, introduce some new features and propose a combination of feature sets for a real-time classification system while maintaining comparatively high accuracy. To identify the relevant set of features for this problem domain, trying all combinations of features is not feasible. Hence, features are categorized into intensity, region, graph, histogram, texture, and shape adaptive features. Region features are extracted using binarized images generated by Otsu’s [29], green percentile thresholding, and morphological thresholding. The effects of normalization, feature reduction with principle components analysis (PCA) [30], and feature selection using random forest classifier are also evaluated. The time required to extract feature categories is computed and an estimated time of feature extraction is provided for feature category combinations. In this way, research groups may ignore some feature groups since they may not have significant effect on the accuracy. This also enables research groups to select features according to their hardware setups for real-time analysis.

In this research, we consider a 9 point scoring system (Hampton’s scores) to classify protein crystallization trial images using hierarchical classification. The first-level of classification categorizes into non-crystals, likely-leads, and crystals. The total number of subcategories is 10 (one more than Hampton’s scale to include a category for unclear bright images). The complete feature set contains around 300 features. Feature sets are categorized into 10 groups and evaluate classifiers exhaustively on all combinations of these feature groups. A random forest (RF), naïve Bayesian (BYS), support vector machine (SVM), decision tree (DT), and neural network (NN) classifiers are utilized in these experiments. Moreover, we investigate the performance of feature selection and normalization. Our goal is to identify a minimal set of feature sets that will achieve good accuracy for real time applications. Around 8,624 experiments (different combinations of feature categories, binarization methods, feature reduction/selection, normalization, and crystal categories) are conducted and a summary of the experimental results is provided. Our system is able to answer the question: “what set of features satisfies a minimum accuracy measure *m* within time *t*?”.

## Materials and methods

### Image categories

**Fig. 2:**
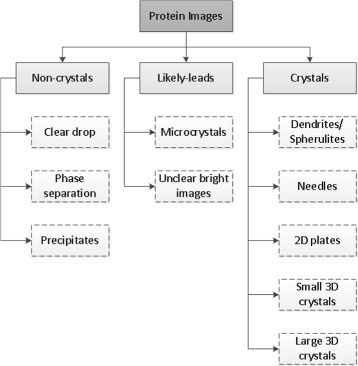
Image hierarchy

Hampton’s scheme defines a scoring system having a range of 9 outcomes for a crystallization trial. In this study, we add one more category to include unclear bright images. Figure 2 shows the hierarchical categories of the protein crystallization images in this paper. In the first level, the crystallization trial images are classified into three categories: non-crystals, likely-leads, and crystals. Description of these categories and their sub-categories is presented next.

#### Non-crystals

Images in non-crystal category do not have any crystal objects. This category consists of images in the following phases: clear drop, phase separation, or precipitates.

1. *Clear drop:* This category indicates that the protein remains homogeneous in the solution because of insufficient degree of super-saturation or because the growing has just started in the metastable phase. Figure 3(a-b) shows some sample images in this category.

**Fig. 3:**
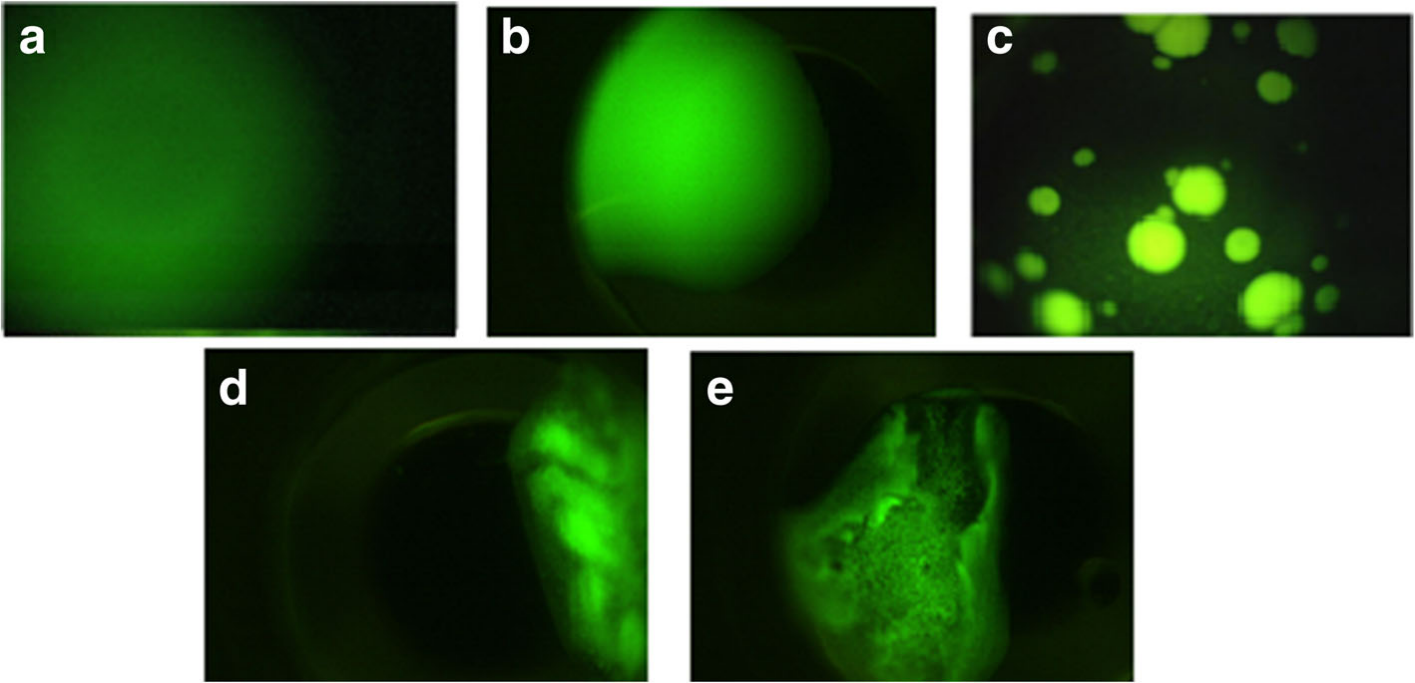
Sample images in non-crystal category **a**-**b**) Clear drops, **c**) Phase separation, **d**-**e**) Precipitates
2. *Phase separation:* Phase separation images occur when the concentration of the protein is too high such that it causes the separation of the protein from the entire solution. This may also occur when one of the solution components separates out from solution, possibly carrying the protein with it. Thus, this phase results in liquid drops, and it is also called oiling out. Phase separation droplets may be numerous and small or few and large depending upon solution conditions and time. Figure 3(c) provides a sample image in protein crystallization, and when it concentrates the protein it has been known to be a source of crystal nucleation.
3. *Precipitates:* When the degree of supersaturation is very high, aggregate precipitates appear in the solution. These images generally have cloud-like shape as shown in Fig. 3(d-e).

#### Likely-leads

This category consists of images corresponding to likely-lead conditions, and hence these can be a good starting point for optimizing the crystallization conditions. Birefringent precipitate or micro-crystals would fall in this category. We also include images with high intensity without clear shapes indicating crystals. High intensity might suggest the presence of crystals. However, as the shapes of the objects do not match to crystal structures, they are grouped into the likely-lead category. This category is the fall back position for missed crystal leads, saving those results for subsequent evaluation by the experimenter.

1. *Microcrystals:* This category consists of images with granular crystal forms. Some representative images are shown in Fig. 4(a-c).

**Fig. 4:**
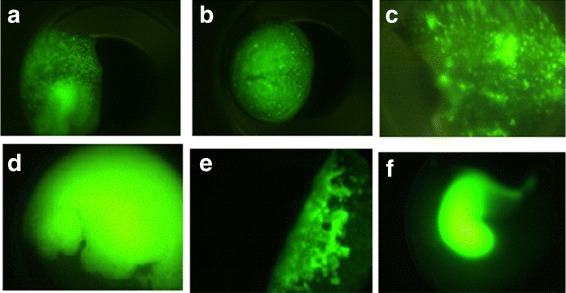
Sample images in likely-lead category **a**-**c**) Microcrystals **d**-**e**) Unclear bright images
2. *Unclear bright images:* This category consists of images which have very high intensity without any crystal objects visible. These images need to be reviewed by an expert. Some representative images are shown in Fig. 4(d-f).

#### Crystals

This category includes images having clear crystal objects. The crystals can have different shapes and sizes such as needles, spherulites, plates, or 3D crystals.

1. *Dendrites/Spherulites:* The images in this category are non-faceted crystalline outcomes, such as urchins, dendrites, spherulites, etc. These show high fluorescence intensity without the proper geometric shapes expected for a faceted crystal. Some representative images are shown in Fig. 5(a).

**Fig. 5:**
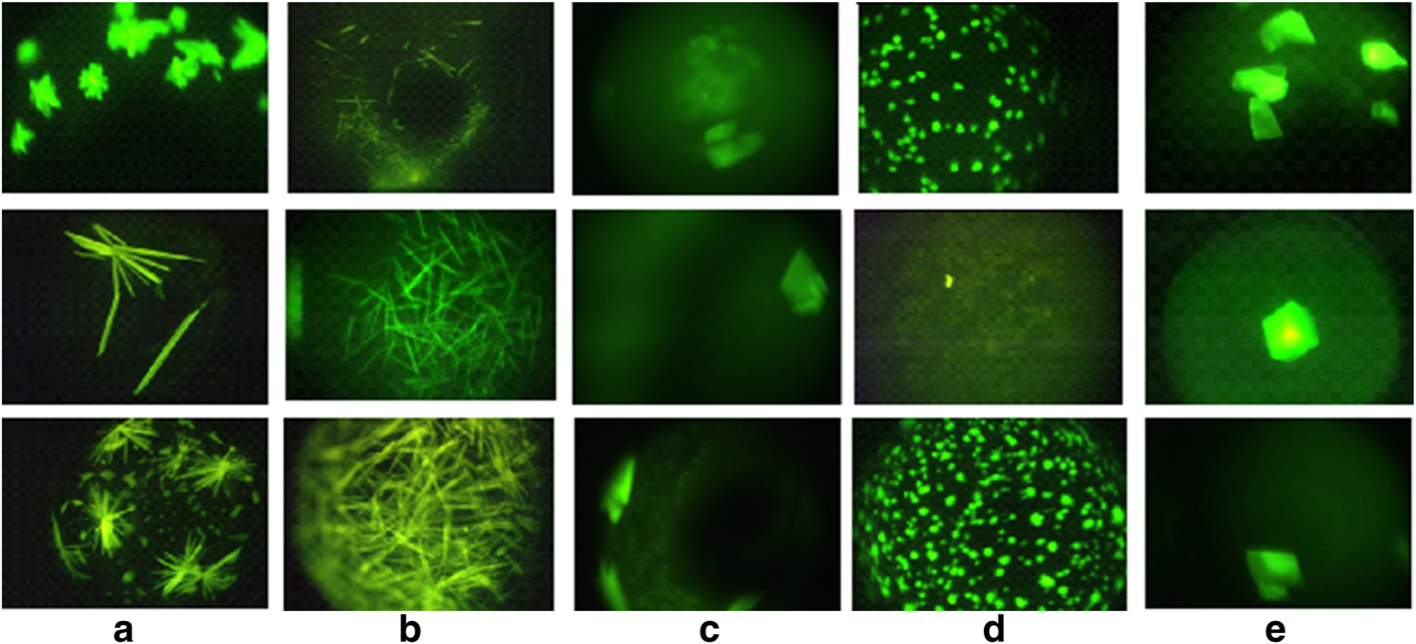
Sample images in crystal category **a**) Dendrites/Spherulites **b**) Needles **c**) 2D plates **d**) Small 3D crystals **e**) Large 3D crystals
2. *Needles:* Needle crystals are differentiated from rods by their having pointed ends, looking like needles. These crystals can appear alone or as a cluster in the images. The overlapping of multiple needle crystals on top of each other makes it difficult to get the correct crystal structure for these images. Figure 5(b) shows some sample images in this category.
3. *2D plates:* 2D plate images have quadrangular shapes and they may have any size in the image. The distinctive characteristic of this category from 3D crystals is that 2D plates have less intense regions than 3D crystals.
  For some specific cases, it is hard to detect or observe edges of those objects due to noise, poor illumination and focusing problems. Figure 5(c) shows some sample images in this category.
4. *Small 3D crystals:* This category contains small sized crystals. These crystals have 3-dimensional shapes. They can appear alone or as a cluster in the images. Because of their small size, it is difficult to observe the geometric shapes expected in crystals. Moreover, crystals may be blurred because of focusing problems. Figure 5(d) shows some sample images in this category.
5. *Large 3D crystals:* This category includes images with large crystals with 3-dimensional shapes. Depending on the orientation of protein crystals in the solution, more than one surface may be visible in some images. Figure 5(e) shows some sample images in this category.

### Data

The images are collected using the Crystal X2 by iXpressGenes, Inc. This is a fluorescence based microscopy system for scanning protein crystallization screening trial plates. All the images are hand scored by an expert according to Hampton’s scale. Table 2 provides the distribution of our dataset into different categories. Our data set includes a total of 2756 images composed of 1600 non-crystal images, 675 likely-lead images, and 481 crystal images. The image resolution is 320×240, reduced from the camera resolution of 2560 x 1920. Some images were difficult to assign a sub-category due to blurriness, illumination problems, significant high intensity in the image, and presence of crystals at different phases. Because of this, we added *doubtful* sub-category in each category, and the images with ambiguous sub-category were assigned to these *doubtful* sub-categories. Doubtful images are used for training at the first level, but these images are discarded while building a training model for sub-category classification.

**Table 2:** Dataset image distribution

Category	Total images	Sub-category	No. of images	Percentage
Non-crystals	1600	Clear drop	1273	46.19%
		Phase separation	1	0.04%
		Precipitate	204	7.4%
		Doubtful	122	4.43%
Likely-leads	675	Micro-crystals	122	4.43%
		Unclear bright images	369	13.39%
		Doubtful	184	6.68%
Crystals	481	Dendrites/Spherulites	63	2.29%
		Needles	153	5.55%
		2D Plates	8	0.29%
		Small 3D crystals	129	4.68%
		Large 3D crystals	35	1.27%
		Doubtful	93	3.37%
		Total	2756	

### Feature normalization, reduction and classification techniques

This study investigates various factors that may affect the classification performance of protein crystal images. Data preprocessing may help to improve the performance of knowledge discovery from the data set. Data preprocessing may involve application of data reduction and data transformation methods. To evaluate data reduction, a random forest feature selection with mean decrease in accuracy (*M* *D* *A*−*R* *F*) [31] method was applied. Normalization of feature vectors was also considered as some classifiers are sensitive to the ranges of features.

Individual effects of z-score normalization, PCA feature reduction and random forest feature selection methods were examined. Then various state-of-art classification methods are employed in order to get benefit from different types of classifiers in the literature such as probabilistic, categorical, and ensemble classifiers.

#### Feature normalization with z-score

Data values are measured in different scales or ranges since they have different meanings. Some classification techniques suffer from range differences because the distance metrics are highly sensitive to data range. In order to eliminate this negative effect, normalization maintains a similar range for all data by mapping the data to a pre-defined range or utilizing the mean and standard deviation of the data. Some classifiers benefit from normalization significantly (such as neural networks), while some of them are not affected from range differences (such as naïve Bayesian and decision trees). Z-score normalization was employed to evaluate the effects of normalization. For this, the data is normalized with respect to its mean (*μ*_*v*_) and standard deviation (*σ*_*v*_). The new value (v’) of original data (v) is calculated as in (1).

1 $$  v' = \frac{v - \mu_{v}}{\sigma_{v}}  $$

#### Feature reduction with *PCA* and *MDA*−*RF*

It is possible to have a high number of features to represent a sample in classification problems. However, some of these features may not be informative enough and can be eliminated without any (or with minor) loss of accuracy. Some of them may be highly correlated or some of them might be measured with high noise. In such cases, data reduction techniques are offered to eliminate these useless features. PCA is one of the widely accepted techniques to reduce dimensionality [30]. In simple terms, PCA transforms complete dataset to a new subspace where every dimension is connected to an eigenvalue. The new feature corresponding to the largest eigenvalue represents the most informative feature. Using this idea, a subset of the most descriptive eigenvectors (or principal components) can be selected and rest of them can be eliminated. The original dataset is transformed into a lower dimensional space using this subset of eigenvectors where a smaller size feature vector represents the same sample.

Another common way to reduce the size of data is feature selection. To evaluate feature selection, in this study, we preferred to use mean decrease in accuracy (MDA) algorithm [31] in random forest classifier. MDA assigns rankings to the features by randomly permuting the values of each feature and measuring the change in mean error.

#### Classification techniques

Classification results are highly dependent on several factors such as data type or distribution. In the literature, different classifiers are offered for different factors. In this study, 5 different classifiers were examined to determine the best classifier for this particular problem domain. The selected classifiers are described below.

1. *Decision Tree (DT):* Decision tree is a rule-based classifier that utilizes a tree-based graph of features to decide the class of a sample. In the training stage, a tree structure is constructed where internal nodes represent features and leaf nodes have class labels. In the testing stage, the test sample is classified by reaching the leaf node from the feature hierarchy of the tree. The decision trees are effective on categorical data types. It requires relatively less time to construct a training model (tree) and testing is also quite fast once the tree is induced [32].
2. *Random Forest (RF):* Random forest is an ensemble type classifier that comprises many decision tree classifiers (weak classifier). In the training stage, every decision tree is constructed based on randomly selected samples (bootstrap). Remaining samples (out-of-bag) are used in the testing stage. While constructing a decision tree, not all features are used. A feature subset is also selected randomly. For the final decision, results of all decision trees are combined based on a voting mechanism [32]. In this paper, Matlab code was used for RF which is based on algorithm by Leo Breiman et al.^2^ [33]. We set the number of trees for the random forest classifier as 500. The square root of the total number of features is selected as the number of candidate features at one node of a decision tree [34].
3. *Support Vector Machines (SVM):* Support Vector Machine is a binary supervised classification method. In the training stage, a decision surface (hyperplane) is determined based on boundary samples called *support vectors*. SVM tries to find the optimal hyperplane that maximizes the margin between the two classes. If the data is not linearly separable, SVM can be applied by transforming the input data to high-dimensional feature spaces using kernel functions [32].
4. *Naïve Bayesian Classifier (BYS):* BYS is a probabilistic classifier technique that decides the class of a sample by providing the probability of its membership to the classes. The class with the highest probability is predicted as the result class. In BYS, the features of the data samples are assumed to be independent from other features. This assumption simplifies building a training model. The training stage is fast and classification is independent from the range of the feature values [32]. Also, BYS is considered to be robust to the noisy samples.
5. *Artificial Neural Networks (NN):* Artificial Neural Networks is a supervised classification technique that is composed of interconnected nodes (neurons). Neurons can be organized in layers depending on the complexity of the problem. It tries to learn the weights of the connections between input and output neurons to minimize the error of classification as new data are evaluated in the training stage. NN is commonly used technique for various classification problems such as autonomous vehicle driving, speech recognition, face recognition, etc. [32, 35]. In this study, we use MATLAB built-in neural network toolbox with two layers. The hidden layer has *n*−1 nodes where *n* is the number of features in the dataset.

### Image processing

Automatically determining the phase of crystallization trial images is a complex process and requires sophisticated algorithms to extract features related to the shape and size of objects in an image. Different image processing techniques are applied to the original images and then image features are extracted from several stages of these steps.

For the notations in the subsequent subsections, assume that 1) *I* represents an image of size *h* x *w*, 2) *I*(*x*,*y*) represents the pixel at location (*x*,*y*) where 1≤*x*≤*h* and 1≤*y*≤*w*, 3) *I*_*G*_ is the green component of image *I*, 4) *I*_*gray*_ is the gray-level image of image *I*, 5) *B*_*m*_ represents the binary image of image *I* using method *m*, and 6) *E* represents edge image using edge detection methods such as Sobel or Canny.

#### Image thresholding

The objective of image thresholding is to simplify the image analysis by separating the foreground pixels from the background. Thresholding is often the first step in image analysis. Obtaining a good binary image is very critical in image analysis because any error in the binary image will get propagated into further processing steps. Numerous image binarization techniques have been proposed in the literature. However, as we discussed in our previous work [36, 37], there is not a single technique which works well in all image domains. In this paper, 3 different image binarization techniques are investigated: Otsu’s threshold [29], green percentile image binarization [28] with two percentiles, and morphological thresholding [38].

*Otsu’s thresholding.* Otsu’s method [29] iterates through all possible threshold values and calculates a measure of spread of the pixel levels in foreground or background region. The threshold value (*τ*_*o*_) for which the sum of foreground and background spreads is minimal is selected. The binary image ($B_{otsu} = \xrightarrow {\tau _{o}}(I_{gray})$) is constructed by applying this threshold to the image.

*Green percentile thresholding.* This method utilizes green color component of image pixels for thresholding. Let *τ*_*p*_ be the intensity of green component such that the number of pixels in the image with green component below *τ*_*p*_ constitute *p* *%* of the pixels. For example, if *p*=90*%*, *τ*_90_ is the intensity of green such that 90% of the green component pixels will be less than *τ*_90_. Image binarization is then done using the value of *τ*_*p*_ and a minimum gray level intensity condition *τ*_*min*_ = 40. All pixels with gray level intensity greater than *τ*_*min*_ and having green pixel component greater than *τ*_*p*_ constitute the foreground region while the remaining pixels constitute the background region. As the value of *p* goes higher, the foreground (object) region in the binary image usually becomes smaller. For the given value of *p*, the method is represented as *G*_*p*_. For example, *G*_90_ is the green percentile thresholding method with *p*=90*%*. *G*_90_ and *G*_99_ are applied for binarization of images in our experiments.

*Morphological Thresholding.* In this method, the images are binarized based on mathematical morphological operations along with some preprocessing methods. The method can be summarized as follows:

1. Apply image-opening function to get background surface: This is one of the basic mathematical morphological operations as in (2):
  2 $$  A \cdot B = (A \ominus B) \oplus B  $$
  where ⊖ and ⊕ denote erosion and dilation, respectively. The basic effect of the erosion operator on a binary image is to erode away the boundaries of regions of foreground pixels. In other words, after this operation the foreground regions generally shrink based on a structure element. On the other hand, after dilation operation the foreground regions generally expand.
2. Subtract background image from grayscale image.
3. Adjust pixel intensities to enhance the images: Contrast stretching is applied to increase the contrast between foreground and background.
4. Binarize the grayscale image using Otsu’s thresholding method.
5. Apply image opening function to generate the final binary image.

#### Region segmentation

Connected component labeling [39] is applied on binary images to extract high intensity regions or blobs. The binary image can be obtained from any of the thresholding methods. Let *O* be the set of the blobs in a binary image *B*, and *B* consists of *n* number of blobs. The *i* ^*th*^ largest blob is represented by *O*_*i*_ where 1≤*i*≤*n* and *a* *r* *e* *a*(*O*_*i*_)≥*a* *r* *e* *a*(*O*_*i*+1_), ∀*i*. Each blob *O*_*i*_ is enclosed by a minimum bounding rectangle (MBR) centered at ($m^{i}_{x},m^{i}_{y}$) having width *w*_*i*_ and height *h*_*i*_. *Ω*_*i*_ represents the skeleton of blob *O*_*i*_. We focus on extracting features related to the shape and size of the top largest blobs.

### Feature extraction

To analyze the classification performance for different features, the image features are grouped into different groups such as intensity features, histogram, texture, region, graph, and shape adaptive features. Feature extraction stage was done mostly using MATLAB programming language. However, in a small portion of the implementation, C# was also used.

#### Intensity features

Features related to intensity distribution in an image can provide a basic feature set to categorize images into different categories. In general, the images consisting of crystals have high illumination compared to the images without crystals. Using the grayscale image *I*_*gray*_, we extract the 6 image intensity features (average image intensity, minimum image intensity, maximum image intensity, standard deviation of intensity, Otsu’s threshold intensity, and threshold effectiveness metric) listed in Table 18 of Appendix.

#### Histogram features

The intensity histogram of an image provides a graphical representation of the image intensity distribution. The histogram provides information about the distribution of all pixel values or group of values in the image. For the fluorescence based images, the green color channel carries the most information. Therefore, the intensity values in this channel are used to compute the histogram features. The number of bins was determined as 256 (between 0 and 255) for each green channel level. Histogram for the green level is defined as:

3 $$  H[\!k] = \sum\limits_{p=1}^{w} \sum\limits_{q=1}^{h} \left\{\begin{array}{ll} 1 & \text{if \(I_{G}(p,q)=k\)} \\ 0 & otherwise \end{array}\right.  $$

Green Level Co-occurrence Matrix (GLCM) is a matrix of distribution of co-occurring values of green level intensity at a given offset *Δ*_*x*_, *Δ*_*y*_ [40]. GLCM matrix *P* using the green color channel is defined as in (4).

4 $$  P_{\Delta x, \Delta y} (i,j) = \sum\limits_{p=1}^{w-\Delta x} \sum\limits_{q=1}^{h-\Delta y} \left\{\begin{array}{ll} 1 & \text{if \(I_{G}(p,q)\) = i and \(I_{G}\)(p+\(\Delta\)x, q+\(\Delta\)y) = j} \\ 0 & otherwise \end{array}\right.  $$

With (*Δ*_*x*_, *Δ*_*y*_) as (1, 0), (0,1) and (1,1), we obtain 3 GLCMs, represented as *P*_1_, *P*_2_, and *P*_3_, respectively. Using green channel image *I*_*G*_, intensity histogram *H* and *GLCM*s *P*_1_, *P*_2_, and *P*_3_, we extract the 21 image features listed in Table 19 of Appendix. The average intensity, standard deviation, skewness, kurtosis, and entropy measure are the image features related to intensity distribution. GLCM auto-correlation is a measure of linear dependence between the elements of co-occurrence matrix with offset of *Δ* *m* and *Δ* *n*. The GLCM auto-correlation *g*_*k*_ with offset (*Δ*_*m*_, *Δ*_*n*_) using GLCM *P*_*k*_ is defined as in (5).

5 $$  {g_{k}}_{\Delta m, \Delta n} = \frac { {\sum\nolimits}_{i=\Delta m}^{255}{\sum\nolimits}_{j=\Delta n}^{255}{ P_{k}(i,j)*P_{k}(i-\Delta m, j-\Delta n)}} {{\sum\nolimits}_{i=\Delta m}^{255}{\sum\nolimits}_{j=\Delta n}^{255} {max (P_{k}(i,j), P_{k}(i-\Delta m, j-\Delta n))^{2}}}  $$

Using *P*_1_, *P*_2_, and *P*_3_ GLCMs, and (*Δ* *m*,*Δ* *n*) as (1, 0), (0,1) and (1,1), we obtain 3*3 = 9 GLCM auto-correlation features.

Image auto-correlation is defined as the measure of linear dependence between pixels of the image with offset of *Δ* *m* and *Δ* *n* and computed as in (6).

6 $$  ac_{\Delta m, \Delta n} = \frac { {\sum\nolimits}_{i=\Delta m}^{255}{\sum\nolimits}_{j=\Delta n}^{255}{I_{G}(i,j)*I_{G}(i-\Delta m, j-\Delta n)}} {{\sum\nolimits}_{i=\Delta m}^{255}{\sum\nolimits}_{j=\Delta n}^{255}{(I_{G}(i,j))^{2}}}  $$

We extract 3 image auto-correlation features using (*Δ* *m*, *Δ* *n*) as (1, 0), (0,1) and (1,1). The green color channel of the image is used as the input. Similarly, the power spectrum is calculated using *P*_1_, *P*_2_, *P*_3_, and *I*, and the magnitude is used as the image feature.

#### Texture features

A texture is a set of texture elements or texels occurring in some regular pattern. In this study, a total of 23 texture features are employed, collected from 3 different studies ([40–42]), and MATLAB built-in functions [43]. The list of features is provided in Table 20 of Appendix. Since we have generated 4 angular GLCM matrices for texture analysis, 4 values are computed for each of 23 features in Table 20 Appendix leading to 4∗23=92 values. By taking the mean and the range of the 4 values per feature, the number of features is reduced to 46.

#### Region features

Image thresholding separates the foreground and background in the image. By thresholding the protein crystal images, crystals are expected to be distinguished as foreground objects. Although other non-crystal objects might also appear as the foreground, features from the binary images can provide important information about the content of an image. Similarly, features related to the shape and size of individual objects are useful to categorize the images into different categories.

Using the gray level image *I*_*gray*_ and binary image *B*, the 7 global binary image features (the number of white pixels in *B*, foreground average intensity, standard deviation of foreground intensity, background average intensity, standard deviation of background intensity, number of blobs, and image fullness) listed in Table 21 of Appendix are extracted. More information about the objects is obtained by extracting features related to intensity statistics and shapes of the individual blobs. 9 blob features (average intensity, standard deviation of intensity, number of pixels, number of white pixels, perimeter, convex hull area, blob eccentricity, blob extent, and equivalent circular diameter) are extracted for each of the top *k* largest blobs. Table 22 of Appendix provides the list of 9 blob features. If the number of blobs *n* is less than *k*, the value 0 is used as the feature value for the blobs *O*_*n*+1_..*O*_*k*_. Since a single technique may not always provide correct binary image, we apply 4 different image binarization (Otsu, *G*_90_, *G*_99_, and morphological thresholding), and use these images to extract region based image features. From each binary image, 52 (7 + 5*9 = 52) image features are obtained for the 5 largest blobs (i.e., *k*=5). Region *Otsu*, Region *G*_90_, Region *G*_99_, and Region *Morph* represent the features obtained using Otsu, *G*_90_, *G*_99_, and morphological thresholding methods, respectively.

#### Graph features

The structure of an object as a graph has significant importance in image analysis since it defines the boundaries of an object in the image. We apply edge detection followed by some post-processing steps to extract features that are useful to define the shapes of objects [44]. In addition, Hough line transform is applied to extract line features. Table 23 of Appendix provides graph related features.

#### Shape adaptive features

Shape-adaptive Discrete Cosine Transform (SA-DCT) is a 2D Discrete Cosine Transform (DCT) method for coding arbitrarily shaped image segments [45]. Image coding can be applied either to region of interest (blobs) or the background region. In this study, we apply SA-DCT on the top largest blobs. Table 24 of Appendix provides the list of image features extracted from each blob after applying the SA-DCT. Otsu’s thresholding is applied to obtain the binary image. SA-DCT is then applied on top 5 largest blobs. Thus, 15 DCT features are obtained from an image. If a binary image contains less than 5 blobs, 0 is assigned to all feature values of missing blobs.

## Results

There are a number of difficulties for classifying crystallization trial images as mentioned in the introduction. First, there are many categories (9 categories according to Hampton’s scale) to classify with high intra-class diversity. As the number of categories increases, developing a reliable classification model becomes more difficult. Second, labeling the data is difficult due to the temporal transition between categories and the presence of multiple types of crystals in images. Third, the low percentage of representation of critical categories gives bias to more populated but less important categories. To overcome these problems, a 2-stage classification was considered that divides the classification problem into 3-class classification (non-crystals, likely-leads and crystals) at the first level, and classification into sub-categories in the second level as shown in Fig. 2. To balance the data distribution, all available data from critical categories was used while reducing the images from frequently occurring image categories. For time analysis, the time to extract each feature set was computed. The classification results based on overall accuracy and sensitivity of critical categories were ranked. 5- and 10-fold cross validation was used for measuring the accuracy in different tests. Accuracy measures along with time analysis for classification help to select the best feature sets for real time stand-alone computing system.

### Time to extract features and classify

Feature extraction was run on a system with Intel Core i7 2.4 GHz CPU, and 12 GB RAM memory. Our image feature extraction routines are implemented using Matlab 2013b. Some feature extraction modules were implemented using C# on Visual Studio 2012. Classification of data was accomplished using Matlab. Table 3 provides a summary of feature extraction timings for different feature sets. Most of the features can be extracted in less than half a second. The set of DCT features is the most computationally expensive feature set since it took around 25.5 s to extract DCT features on the average per image. This may be due to inefficient shape adaptive DCT implementation. However, we still use it in our experiments to observe its benefit to the accuracy of the classification. Texture and intensity features can be extracted quite fast in about 0.037*s* and 0.052*s*, respectively.

**Table 3:** Computation time for feature extraction

Feature group	Description	No of features	Avg time per feature	Avg time per image
Intensity	Intensity features	6	0.009	0.052
Region Otsu	Region features using Otsu	52	0.005	0.258
Region *G*_90_	Region features using *G*_90_	52	0.010	0.495
Region *G*_99_	Region features using *G*_99_	52	0.004	0.193
Region Morph	Region features using morph thresh	52	0.006	0.311
Graph	Hough features and edge features	13	0.022	0.284
Hough	Hough features only	2	0.049	0.097
Texture	Texture features	46	0.001	0.037
Histogram	Histogram features	21	0.009	0.178
DCT	DCT features	15	1.709	25.639

In the timing analysis, we calculate the total time using individual extraction times in Table 3, when a combination of feature sets is selected. For example, if the feature set combination involves intensity, region *G*_90_, and texture features, the total time to extract these feature sets combination is computed as 0.052+0.258+0.037=0.347*s*.

We also need to include the time to classify provided feature sets. We have computed the time to classify using random forest classifier as it provided better accuracy than other classifiers (to be explained later in the following sub-section). The random forest classifier also provides an upper-bound for classification time as it is more complicated than other compared classifiers in terms of evaluation due to the number of decision trees involved. Random forest takes roughly 0.361 s to test all our features, which is less than a half second for the complete set. If the feature set composed of intensity, region *G*_90_, and texture features is classified using random forest classifier, the time to extract features and classify is computed as 0.347+0.361=0.708*s*. For the hierarchical classification, new features may need to be extracted for the other levels, and again a classifier needs to be applied for these levels. Hence, the timings for other levels should be added as well.

### Experiments

In this study, the experiments are designed in an exhaustive manner to be able to evaluate effectiveness of different factors for classification of protein crystal images. Different feature sets, classifiers, normalization and feature reduction techniques are considered. Experiments are carried out for all possible cases, and the performance is calculated for each case. The goal is to determine the best condition (feature set/classifier/transformation tuple) that can yield the highest accuracy on protein crystallization images. The selection of features for hierarchical classification is provided in “Evaluating features for hierarchical classification” section. The results with respect to the time complexity as real time processing, one of the main concerns in our system, are evaluated. A total of **8624** experiments are carried out to test 9 major objectives, listed in Table 4. According to the table, Exp. IDs from “1” through “4” represent the first level experiments that are described in “First level (3-class) classification” section, and Exp. IDs from “5” to “7” describe the second level experiments explained in “Second level classification” section. In addition, Exp. IDs “8” and “9” correspond to timing calculation of the experiments explained in “Time to extract features and classify” section.

**Table 4:** List of classification experiments

Exp ID	Tasks	No. of experiments ^3^
1	Run all classifiers for 511 feature set (5 classifiers with/without normalization)	2 * 5 * 511* 1 = 5110
2	Run the best classifier 5 times and take the average for the best 70-feature set (RF)	1 * 1 * 64 * 5 = 320
3	Run classifiers PCA for 10,20,..,50 features	1 * 5 * 5 * 2= 50
4	Run classifiers using RF feature selection (10,20,...,50)	1 * 5 * 5* 2 = 50
5	Run BYS, DT and RF (with and without normalization, with graph features) for crystal sub categories	2 * 3 * 511 * 1 = 3066
6	Run RF, DT and BYS classifiers with and without normalization for likely-lead subcategories	2 * 3 * 1 * 1 = 6
7	Run RF, DT and BYS classifiers with and without normalization for non-crystal subcategories	2 * 3 * 1 * 1 = 6
8	Calculate training and testing time of the random forest for the largest feature	1 * 1 * 1 * 5 = 5
9	Calculate timings for feature extraction of an image	1 * 1 * 11 *1 = 11
	Total number of experiments	8624

#### Evaluating features for hierarchical classification

We have started our experiments by classifying protein crystallization trial images into the categories of the first level. Analyzing pixel intensities was generally enough for the first level classification. Once we have obtained good results with the first level, we have applied sub-category classification for each category of the first level. Ideally, it would be good if the feature set that works great for the first level also works best for the second level. We do not restrict ourselves with the optimal feature set of the first level for conducting experiments of the second level. For further sub-category classification, we firstly test the performance with the same feature set. If the same feature set provides reasonable performance, there would be no need to extract any more features. However, if the accuracy of subcategory classification is not satisfactory, we run all combinations of feature sets for the subcategory as well.

We have used intensity (Table 18 Appendix), histogram (Table 19 Appendix), texture (Table 20 Appendix), region (Tables 21 and 22 Appendix), Hough (Table 23 Appendix), and shape adaptive (Table 24 Appendix) features for the first level classification. When we were working with an expert, we have realized the expert was also checking the boundaries of crystal regions to actually identify crystals. Using ’Hough’ features did not provide satisfactory results for crystal sub-classification. We thought that adding edge features (Table 23 Appendix) in addition to Hough features would improve the classification accuracy. The main factor for adding this additional set is the diverse set of images in crystal categories (Fig. 5): dendrites/spherulites, needles, plates, small 3D and large 3D crystals. Later we observed that graph features (Table 23 Appendix) turned out to be important for crystal sub-classification.

#### First level (3-class) classification

For the first level of classification, we ran 5110 experiments for all possible feature sets with and without normalization on 5 different classifiers (Exp. ID 1 in Table 4). We have 9 different feature sets as mentioned above. Based on those features, 2^9^−1=511 different combinations of feature sets were generated for the first level classification. For the first level classification, only Hough features of the graph feature set in Table 23 were utilized rather than the complete graph feature set. After analyzing the results of Exp. ID 1, the best 64 feature sets were selected that provided the highest accuracy. Using the selected feature sets, the experiments were rerun 5 times and the average was taken to ensure that the results are consistent (Exp. ID 2 in Table 4). In addition to these experiments, the effects of feature reduction and selection methods on the classification performance were investigated. PCA was applied to the complete feature set (excluding 11 edge features which are added later for crystal sub-categories in Table 23) by reducing from 298 features to 5 feature subsets (10, 20, 30, 40, and 50 features). Later, we ran the 50 experiments (Exp. ID 3 in Table 4). Similarly, random forest feature selection algorithm was applied to reduce the features (10, 20, 30, 40, and 50 features) (Exp. ID 4 in Table 4) similar to the PCA experiments. Then 50 new experiments were run for new feature sets. Totally 5530 experiments were carried out for the first level of classification.

*Accuracy Measures.* To evaluate the correctness of the classification four measures: accuracy, probabilistic accuracy (Pacc) [46], sensitivity, and adjusted sensitivity were evaluated. Let matrix *C* represent the *N*×*N* confusion matrix for an N-class problem. The value *C*_*ij*_ refers to the number of items of class *i* predicted as class *j*. For the first-level (3-class) classification, adjusted sensitivity is calculated as in (7).

7 $$  adjusted\text{} \,\,sensitivity = \frac {{\sum\nolimits}_{i=2}^{i=3} C_{2i} + C_{3i}} {{\sum\nolimits}_{i=1}^{i=3} C_{2i} + C_{3i}}  $$

Here, classes 1, 2 and 3 represent non-crystals, likely-leads and crystal categories, respectively. The adjusted sensitivity does not penalize if crystals are classified as likely-leads since experts analyze the likely-lead category as well.

*Best Performing Feature Sets.* Table 5 shows the best 10 results of 5110 experiments in Exp. ID 1 in descending order with respect to the accuracy measure. Here, the highest accuracy result (96.3%) is achieved by applying random forest classifier on the following normalized feature sets: intensity features, region features using Otsu, region features using *G*_99_, and histogram features. As can be seen in the table, the other results are also satisfactory as much as the first one. Note that the DCT features require significant extraction time and provide very little or no contribution to the overall classification performance. Therefore, in the second level of classification, we excluded DCT features from the experiments.

**Table 5:** Classification results for preliminary experiment using random forest classifier (Experiment ID 1)

Feature set	Norm.	Acc	Pacc	Sensitivity	Adjusted sensitivity
Intensity, Region Otsu, Region *G*_99_, Histogram	Yes	0.963	0.942	0.867	1
Intensity, Region Otsu, Region *G*_99_, Region Morph, Histogram, DCT	No	0.963	0.942	0.871	1
Intensity, Region Otsu, Region *G*_99_, Hough, Texture, Histogram, DCT	Yes	0.963	0.941	0.863	1
Intensity, Region Otsu, Region *G*_99_, Histogram	No	0.962	0.94	0.881	1
Intensity, Region Otsu, Region *G*_90_, Region *G*_99_, Region Morph, Hough, Histogram, DCT	No	0.962	0.94	0.867	1
Intensity, Region Otsu, Region *G*_90_, Region *G*_99_, Region Morph, Texture, Histogram	Yes	0.962	0.939	0.865	1
Intensity, Region Otsu, Region *G*_99_, Hough, Histogram, DCT	Yes	0.962	0.939	0.871	1
Intensity, Region Otsu, Region *G*_99_, Hough, Histogram, DCT	No	0.962	0.939	0.869	1
Intensity, Region *G*_99_, Hough, Texture, Histogram	Yes	0.962	0.938	0.861	1
Intensity, Region Otsu, Region *G*_90_, Region *G*_99_, Region Morph, Histogram	No	0.962	0.938	0.861	1

*Re-evaluating the Best Results.* After conducting 5110 experiments the best 64 feature sets were selected to validate the consistency of their high performance. Then, these particular experiments were repeated for these 64 feature sets 5 times and their average performance was calculated. In Table 6, the feature sets along with the accuracies of the best 8 (out of 64) experiments are provided. The set of intensity features, region features using Otsu, region features using *G*_90_, region features using *G*_99_, and histogram features gave the best accuracy (96.1%) using random forest classifier. According to the time analysis, the best feature set can be extracted in 1.080 s. This is not the lowest time in the table, but it is a reasonable time for real time applications.

**Table 6:** Classification results for the best 8 of 64 experiments using random forest classifier

Feature Set	Norm.	Acc	Pacc	Sensitivity	Adjusted sensitivity	Time per image (sec)
Intensity, Region Otsu, Region *G*_90_,	No	0.961	0.938	0.87	1	1.08
Region *G*_99_, Histogram						
Intensity, Region Otsu, Region *G*_90_,	No	0.96	0.935	0.857	1	1.31
Region *G*_99_, Region Morph, Texture, Histogram						
Region Otsu, Region *G*_90_, Region *G*_99_, Histogram	Yes	0.959	0.935	0.861	1	1.028
Region Otsu, Region *G*_90_, Region *G*_99_, Histogram, DCT	No	0.959	0.934	0.852	1	26.668
Region Otsu, Region *G*_99_, Histogram	Yes	0.959	0.934	0.858	1	0.77
Region Otsu, Region *G*_90_, Region *G*_99_, Histogram	No	0.959	0.934	0.859	1	1.028
Intensity, Region Otsu, Region *G*_90_, Region *G*_99_, Texture,	No	0.958	0.934	0.854	1	26.756
Histogram, DCT						
Region Otsu, Region *G*_99_, Histogram, DCT	No	0.957	0.931	0.853	1	26.409

**Fig. 6:**
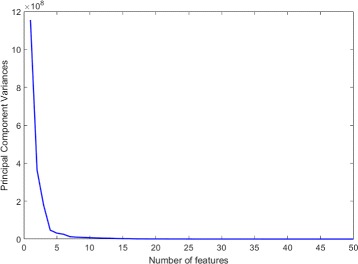
Principal component variances of the best 50 features

*Feature Reduction using PCA.* Feature reduction was also considered to determine its effect on the classification performance. First, we reduced the number of complete feature set using PCA. Five new feature sets (10, 20, 30, 40, and 50 features) were generated that include the most representative ones in the new feature space. For each feature set, the experiments were evaluated using all classifiers with and without normalization (Exp. ID 3 in Table 4). The accuracy measures were calculated and the results are provided in Table 7. The highest accuracy can be reached using 30 or 20 features (with PCA transformation) using random forest classifier after applying normalization. The change in principal component variances with respect to the number of features is shown in Fig. 6. By analyzing Table 7, we can infer that the number of features can be reduced to 20 with a small loss of accuracy (around 3% lower than the best case in Table 5). However, the sensitivity is almost 0.13 lower than the best sensitivity.

**Table 7:** Classification results after feature reduction by PCA

Classifier	# Features	Norm	Acc	Pacc	Sensitivity	Adjusted sensitivity
RF	30	Yes	0.934	0.901	0.740	0.954
RF	20	Yes	0.934	0.905	0.744	0.944
RF	40	Yes	0.931	0.897	0.728	0.948
RF	50	Yes	0.930	0.896	0.719	0.950
RF	50	No	0.928	0.893	0.715	0.940
SVM	50	Yes	0.918	0.870	0.761	0.990
SVM	40	Yes	0.916	0.869	0.763	0.983
SVM	30	Yes	0.910	0.858	0.726	0.985
RF	40	No	0.909	0.880	0.688	0.861
SVM	50	No	0.909	0.858	0.765	0.983

*Feature Selection using Random Forest MDA.* Similar to the feature reduction, the effects of feature selection was also considered in our experiments. To select more reliable features, MDA (mean decrease in accuracy) algorithm in Random Forest was preferred. Five feature sets (having 10, 20, 30, 40, and 50 representative features) were generated. For each feature set, the experiments were evaluated using all classifiers with and without normalization (Exp. ID 4 in Table 4). Similar to the PCA reduction results in Table 7, four accuracy measures were calculated. The results were reported in Table 8. Best results were achieved using 30 features with Random forest classifier after normalizing the dataset. The comparison of the best results in Tables 7 and 8 show that feature selection provides better accuracy than feature reduction in our experiments.

**Table 8:** Classification results after feature selection by Random Forest

Classifier	# Features	Norm	Acc	Pacc	Sensitivity	Adjusted sensitivity
RF	30	Yes	0.960	0.936	0.863	0.998
RF	40	No	0.958	0.933	0.852	0.994
RF	50	Yes	0.957	0.932	0.859	0.996
RF	50	No	0.956	0.930	0.859	0.996
RF	30	No	0.954	0.926	0.834	0.994
RF	30	Yes	0.952	0.925	0.817	0.994
RF	20	No	0.950	0.920	0.832	0.992
RF	20	Yes	0.946	0.915	0.817	0.996
SVM	30	Yes	0.938	0.901	0.854	0.996
SVM	50	Yes	0.934	0.895	0.844	0.996

*Performance of Individual Feature Sets.* Finally, the power of the individual feature sets was investigated. The performance of each feature set was evaluated using all classifiers with and without normalization. Table 9 shows the best results for each feature set. Additional experiments for these results were not performed since Exp. ID 1 already includes these cases. The best results are obtained using the histogram feature sets with accuracy of 90.8*%*.

**Table 9:** Classification performance with individual feature sets

Feature Set	Classifier	Norm	Acc	Pacc	Sensitivity	Adjusted sensitivity
Intensity	ID3	No	0.877	0.836	0.701	0.950
Region Otsu	BYS	Yes	0.751	0.702	0.622	0.915
Region *G*_90_	SVM	Yes	0.864	0.818	0.676	0.944
Region *G*_99_	SVM	Yes	0.882	0.838	0.723	0.944
Region Morph	BYS	Yes	0.738	0.717	0.580	0.994
Hough	SVM	Yes	0.841	0.737	0.235	0.906
Texture	ID3	Yes	0.822	0.778	0.605	0.877
DCT	BYS	Yes	0.691	0.647	0.480	0.775
Histogram	SVM	Yes	0.908	0.852	0.705	0.996

#### Second level classification

We preferred to evaluate the second level classification independently. This helps us analyze and improve the sub-category classification by ignoring the misclassification from the first level. If the classification accuracy of the first level was low, this could have been risky. However, our first level accuracy is 96%, which is reasonably high. In the first level classification, protein crystallization trial images are classified into 3 categories: non-crystals, likely-leads and crystals. In the second level, each of these categories are further classified into sub-categories as shown in Fig. 2. For the first level, the feature set composed of intensity features, region features using Otsu, region features using *G*_90_, region features using *G*_99_, and histogram features (First row in Table 6) provided the best result was determined.

We provide the sensitivity for the highest ranked category in each sub-category. The highest ranked category is precipitates for non-crystals, micro-crystals for likely-leads, and large 3D crystals for the crystals. For two- class classification, if both accuracy and sensitivity are available along with the number of samples in each category, the other sensitivity value could be computed easily.

*Non-crystal classification* Non-crystals are classified into 3 sub-categories: clear drops, phase separation, and precipitates. Phase separation is a relatively rare occurrence. Table 10 provides the classification performance for 3 classifiers (Exp. ID 7 in Table 4) with and without normalization. These experiments are conducted on the best feature set combination for the first-level classification. Normalization is done using z-score normalization. The sensitivity column refers to the sensitivity for precipitates. Random forest provided the best classification performance and normalization did not make any major difference. The classification accuracy is 98% and the sensitivity for precipitates category is 0.91.

**Table 10:** Non-crystal sub-classification

Classifier	Normalization	Accuracy	Pacc	Sensitivity
Naïve Bayes	No	0.88	0.71	0.59
Naïve Bayes	Yes	0.88	0.72	0.68
Decision Tree	No	0.96	0.79	0.85
Decision Tree	Yes	0.96	0.79	0.85
Random Forest	No	0.98	0.81	0.91
Random Forest	Yes	0.98	0.81	0.91

*Likely-lead classification* In the likely-lead category, there are two sub-categories: unclear bright images and microcrystals. The classification performance with 3 classifiers (Naïve Bayes, decision tree and random forest) is provided in Table 11 (Exp. ID 6 in Table 4). These experiments are again conducted on the best feature set combination for the first-level classification. The sensitivity column refers to the sensitivity for micro-crystals. The best performance (92% accuracy) is obtained using random forest classifier without normalization. The corresponding sensitivity for micro-crystals is 0.80.

**Table 11:** Likely-lead sub-classification

Classifier	Normalization	Accuracy	Pacc	Sensitivity
Naïve Bayes	No	0.59	0.62	0.86
Naïve Bayes	Yes	0.58	0.63	0.93
Decision Tree	No	0.87	0.85	0.74
Decision Tree	Yes	0.88	0.86	0.76
Random Forest	No	0.92	0.91	0.80
Random Forest	Yes	0.91	0.89	0.78

*Crystal sub-classification* In the crystal category, there are 5 sub-categories: dendrites/spherulites, needles, 2D plates, small 3D crystals, and large 3D crystals. Crystals have geometric shapes that can be defined by edges. Therefore, edge related features are quite useful to distinguish the crystal sub-categories. For crystal sub-classification, rather than using only Hough features of the graph feature set, the edge features in Table 23 were also included in our experiments to consider the diverse crystal categories. In addition to the selected features useful for the first level classification and non-crystal and likely-lead classification, classification experiments were performed (Exp. ID 5 in Table 4) including graph features described in “Feature extraction” section. Table 12 shows the top 7 classification performances based on the accuracy using random forest classifier. The sensitivity column refers to the sensitivity of large 3D crystals. The feature set of intensity, region features using Otsu’s thresholding, region features using *G*_90_, graph and histograms gave the highest accuracy of 74.2*%*. This feature set can be extracted in 1.267 s. Alternatively, with slightly lower accuracy (74%), the feature set of region using Otsu’s thresholding, region using *G*_99_, graph and histogram features can be generated in less than a second. The fastest feature set (region features using *G*_90_ and graph) with accuracy of 73.5*%* can be generated in 0.779 s.

**Table 12:** Crystal sub-classification

Feature set	Norm	Accuracy	Pacc	Sensitivity	Time (s)
Intensity, Region Otsu, Region *G*_90_, Graph, Histogram	Yes	0.742	0.667	0.909	1.267
Region Otsu, Region *G*_99_, Graph, Texture, Histogram	Yes	0.74	0.684	0.896	0.949
Region Otsu, Region *G*_90_, Region *G*_99_, Graph, Histogram	Yes	0.737	0.658	0.896	1.408
Region *G*_90_, Graph	No	0.735	0.659	0.902	0.779
Intensity, R_ *G*_90_, R_ *G*_99_, Graph, Histogram	No	0.735	0.667	0.896	1.201
Intensity, R_Otsu, R_ *G*_90_, Graph, Histogram	No	0.735	0.657	0.89	1.267
Intensity, Region Otsu, Region *G*_99_, Graph, Histogram	No	0.735	0.682	0.878	0.964

## Discussion

Real-time applications have deadlines to complete specific tasks. Reduction of features is essential for building real-time computing systems. The Crystal X2 microscopy system was used to collect the images of protein crystallization experiments benefiting from trace fluorescence labeling. Trace fluorescence labeling [47] helps to reduce the number of features significantly with respect to systems using white light. Moreover, since trace fluorescence labeling yields high contrast between crystal regions and the background in trial images, image processing can be done in a simple and fast manner. The time to extract features from images and classify them can be reduced significantly. The time between capturing two images of a crystallization well plate using Crystal X2 is around 3 s. To be able to execute image acquisition and classification in parallel, the feature extraction and classification should be less than the transition time. However, there is a trade-off to consider between the best classification performance and minimum time for feature extraction. While extracting less features may be desirable, it may reduce the classification performance. In the discussions below, we only focus on the first level classification and crystal sub-category classification for the second level of the classification since the accuracy of crystal classification is more important than other sub-categories.

*The Best Feature Sets.* Using all features provided almost the same accuracy for the first level as the best feature sets. The best classification performance for the first level (3-class) classification had 96% accuracy and 0.87 sensitivity using region features from Otsu’s, *G*_90_, and *G*_99_ thresholding, intensity, and histogram features. The feature extraction can be completed in 1.08*s* for this feature set. Deep CNN [19] achieved 96.56*%* accuracy for binary classification by missing around 16% of crystals for their data set. Since the accuracy of the first level classification is high (around 96%), the misclassification at the first level should not have a significant effect on the second level. Our system does not misclassify a crystal as non-crystal at the first level (i.e., the adjusted sensitivity is 1). The best classification performance for crystal sub-categories at the second level had 74.2*%* accuracy and 0.909 sensitivity using normalized intensity, histogram, graph features and region features from Otsu’s and *G*_90_ thresholding. This set of features can be extracted in 1.267*s*. On the other hand, by using all features, 69.6*%* accuracy with 0.618 sensitivity for crystal sub-category classification is obtained. Using all features reduced the accuracy and (more importantly) sensitivity significantly for the second level. The sensitivity of classification using all features for crystal sub-categories is unacceptably low.

*Fast Feature Sets.* The fastest feature extraction with the same accuracy for the first level uses normalized histogram features and region features from Otsu’s and *G*_99_ thresholding. This feature set can be extracted in 0.77*s*. The sensitivity of this feature (0.86) is slightly less than the sensitivity of the best feature set (0.87). Since the classification performance of the fast feature set is close to the performance of the best feature set, this set of features can be preferred to reduce the time for classification. For the crystal sub-category classification, the fastest feature set that can extracted with high accuracy has only region features from *G*_90_ and graph features. This smaller feature set has provided 73.5*%* accuracy and 0.902 sensitivity compared to 74.2*%* accuracy and 0.909 sensitivity of the best feature set.

*Comparison of Feature Sets for Hierarchical Classification.* If two levels of classification are run in a hierarchical way, the union of the best feature sets includes intensity, graph, histogram features, and region features from Otsu’s, *G*_90_, and *G*_99_ thresholding. In other words, only graph features are added for the second level of classification. The total time for feature extraction increases slightly from 1.08*s* to 1.373*s*. Note that the time to extract the best feature set was 1.267*s* for the second level classification. If the fast feature sets from both levels are included, the union of feature sets includes histogram, graph features, and region features from Otsu’s, *G*_90_ and *G*_99_ thresholding. For the fast feature sets, the intersection for the first and second levels is empty. The total time to extract features becomes 1.549s. Using fast feature sets for each level did not improve the overall time at all. The union of the best feature sets can be executed faster for the combination of two levels. If the classifier model is run in a hierarchical way, the overall performance in terms of time should be analyzed with respect to the common features between levels.

*Accuracy for Hierarchical Classification using the Best Feature Sets.* We have computed the accuracy of hierarchical classification using the best feature set by applying the random forest classifier. Since we have used 5-fold cross validation for evaluation, we have to make sure that the training samples used for the second level are also used in the training set of the first level. Similarly, the same case applies for the test set. Such selection limits the selection of training set for the first level. We have used doubtful images for sub-categories in training of the first level but not used for the second level. We have performed these new experiments in a retrospective way and there could be some slight differences in datasets and their categorization. Hence, we provide the confusion matrices for these cascaded classification to avoid confusion. Based on our experiments, the accuracies of the first level and second level are 95.46 and 92.79%, respectively. The overall accuracy of the hierarchical classification is 89.22%. The confusion matrix of both levels is provided in Table 13. The confusion matrix for the first level is provided in Table 14. The confusion matrices for non-crystals, likely leads and crystals are provided in Tables 15, 16 and 17, respectively. In the confusion matrices of the second level, “*” indicates incorrect classification samples in the first level.

**Table 13:** Confusion matrix of hierarchical classification (FL: the first level, SL: the second level)

	SL=True	SL=False
FL=True	2103	147
FL=False	84	23

**Table 14:** Confusion matrix for the first level

		Actual		
	Class	0	1	2
Prediction	0	1474	1	1
	1	2	461	73
	2	2	29	314

**Table 15:** Confusion matrix for non-crystal classification (*: first level misclassification)

		Actual		
	Non Crystals	Clear drop	Phase separation	Precipitate
*Prediction	Clear drop	1265	0	20
	Phase separation	0	0	0
	Precipitate	8	0	181
	*	0	1	3

**Table 16:** Confusion matrix for likely leads classification (*: first level misclassification)

		Actual	
	Likely Leads	Micro-crystals	Unclear bright images
*Prediction	Micro-crystals	97	14
	Unclear bright images	16	334
	*	9	21

**Table 17:** Confusion matrix for crystal classification (*: first level misclassification)

		Actual				
	Crystals	Dendrites/Spherulites	Needles	2D plates	Small 3D	Large 3D
*Prediction	Dendrites/Spherulites	11	1	0	4	0
	Needles	11	99	1	13	0
	2D plates	0	0	0	0	0
	Small 3D	32	7	2	95	12
	Large 3D	0	0	1	5	21
	*	9	46	4	12	2

*Time to Classify Images.* In these experiments, random forest classifier consistently yielded good accuracy for classifying images at both levels. It took around 0.361*s* to evaluate the largest feature set using random forest classifier. If the time to classify using random forest classifier is included, the following timings provided in parentheses for the following feature sets are obtained: the best feature set for the first level (1.441*s*), the best feature set for the second level (1.628*s*), the fast feature set for the first level (1.131*s*), the fast feature set for the second level (1.263*s*), the union of the best feature sets (2.094*s*), and the union of the fastest feature sets (2.271*s*). Note that for the union of feature sets, the random forest classifier is applied twice (one for each level). These timings are promising for incorporating into real-time stand-alone computing systems. Since Crystal X2 takes around 3 s to move from one well to another well (including the time to move the plate and switching the light source), an option for real-time scoring has been implemented into the Crystal X2 system.

*The Number of Features.* The total number of features used in our experiments is 309. The union of best feature sets had 196 features, which is approximately 36% less than the total number of features. The fast feature set for the first level included 125 features, while the crystal sub-classification had 65 features. If classifiers for the first level and crystal sub-category classification are used independently, this leads to around 60% and 80% reduction of features for the first level and crystal sub-category classification using fast feature sets, respectively.

*Individual Feature Sets.* The individual feature sets were evaluated for the first level. The best classification performance was obtained by applying random forest classifier to normalized histogram features. This yielded 90.8*%* accuracy with 0.705 sensitivity. Intensity features using decision tree provided 87.7*%* accuracy with 0.701 sensitivity. DCT features provided the lowest accuracy of 69.1*%* with 0.48 sensitivity. The performance of histogram features is notable as it uses only 21 features which can be extracted in 0.178*s*. However, its relative low sensitivity (0.705) with respect to the sensitivity of the best feature set (0.87) makes using histogram features alone less desirable.

*Use of Multiple Thresholding Methods.* In the preliminary experiments, none of the thresholding methods produced good binarization consistently for all images in our data set due to challenges mentioned in the introduction. Rather than choosing the best thresholding method among these, region features from all thresholded images were extracted and fed to classifiers. Among thresholding techniques, morphological thresholding did not improve accuracy much and it did not appear in feature sets leading to high accuracy. In other cases, good classifiers generally used region features from the two of the thresholding methods. This shows that classifiers can benefit from a set of thresholding methods if at least one of them provides good separation of the background and foreground.

*Feature Selection and Reduction.* Random forest classifier was used to rank features and PCA for feature reduction. The best accuracy for PCA and feature selection was obtained using 30 features by applying random forest classifier. PCA yielded 93.4*%* accuracy, while feature selection provided 96% accuracy. The sensitivity of PCA is low (0.74) with respect to the sensitivity of feature selection (0.863). The performance of feature selection is remarkable and slightly less with respect to the performance of the best classifier.

*Performance of Classifiers and Generalizability.* Random forest classifier consistently performed better than other classifiers. After observing that random forest is more reliable than other classifiers in Exp. ID 1, the best experimental conditions were repeated in Exp. ID 2 using random forest to validate the consistency of their high performance. Normalization barely affected the performance of random forest classifier. There were cases where normalization slightly lowered the performance. We have performed a small set of experiments to measure generalizability over 5 different test sets of 100 samples. SVM had the best generalizability followed by the decision tree and then by the random forest classifier. However, the generalizability could still be an issue for diverse datasets. Our experiments provide the best set of feature sets for each classifier. The best model may need to be retrained for a larger new dataset. If the best model cannot generalize well, the next best model that could generalize could be selected for actual experiments. Overfitting is possible with random forest classifier if many features are used or too many terminal nodes are allowed while building weak classifiers and the dataset does not cover all possible cases. To avoid overfitting, the number of features or the number of terminal nodes may be reduced for the random forest classifier.

## Conclusion

In this paper, feature analysis was performed for protein crystallization trial images benefiting from trace fluorescence labeling. Trace fluorescence labeling along with feature analysis method helps to enable real-time scoring for the Crystal X2 system. Feature extraction and classification can be completed in around 2 s. For hierarchical classification, it may be reasonable to maximize the common feature sets between levels of classification hierarchy. The best experimental results were obtained using combinations of intensity features, region features using Otsu’s thresholding, region features using green percentile *G*_90_ thresholding, region features using green percentile *G*_99_ thresholding, graph features, and histogram features. Using this feature set combination, 96% accuracy was achieved for the first level of classification to determine the presence of crystals and 74.2*%* accuracy for (5-class) crystal sub-category classification using random forest classifier. The correctness of the first level classification should be given more weight since misclassification at the first level affects the second level. The choice of the fastest feature set for each level does not improve overall time if the set of common features is small or empty.

The use of all features may not only increase the processing time but may also lower the accuracy. Using all features had adverse effect on the crystal sub-category classification. It reduced the accuracy from 74.2 to 69.6*%* and sensitivity from 0.909 to 0.618. The experiments show that protein crystallization classification would benefit from feature reduction in terms of time and accuracy. The histogram auto-correlation features ranked high when a feature selection method was applied. Graph features were included in the best feature sets for crystal sub-category classification. DCT features did not have significant positive impact on the accuracy despite its high computational time. Intensity and region features were generally involved in high accuracy feature sets and ranked high in the results of feature selection method. The random forest classifier provided the best results among classifiers in most cases.

If there is no single thresholding method that works well for all images in the data set, classifiers may benefit from the outcomes of multiple thresholding methods assuming at least one of them produces a good result for an image. The feature sets that yielded high accuracy generally included region features from at least two of the thresholding methods. It was also interesting to observe that the region features from morphological thresholding was not included in the best feature sets.

Our exhaustive method of trying different combinations of feature sets, classifiers, crystallization categories, feature selection/reduction methods and normalization helped us observe overall performance about feature sets with different classifiers. Since we maintained timing for feature sets, this lets us identify the best feature set to achieve a specific accuracy within specific time.

Our experiments have been conducted rigorously and improvements or updates have been made as needed throughout the course of experiments. Such updates include ignoring some unnecessary features, updating some existing features, and adding new features as needed. Our future work has two dimensions: 1) reduce time to classify and 2) improve accuracy/sensitivity. When feature extraction time per feature set was computed, the timings were computed individually. The feature extraction has common intermediate steps among feature sets. For example, if the foreground and background intensities are computed, the overall intensity of the image can be computed from these features without processing the complete image again. The intermediate steps do not need to be executed again if the outputs of intermediate results are stored. Moreover, each feature set may have irrelevant features that may not improve the accuracy. If irrelevant features are eliminated, the time to extract features is reduced as well. To improve the accuracy/sensitivity, images that were not classified correctly should be identified. A new set of features may need to be extracted and analyzed for those images to improve the accuracy. We have not observed a significant advantage of using simpler approaches such as linear discriminant analysis or other ensemble methods, however, they could be tried by identifying best parameter combinations and determined if they improve the overall performance.

**Table 18:** List of intensity features

Symbol	Description	Formulation
*i*_*μ*_	Average image intensity	$\frac {1}{w*h} {\sum \nolimits }_{i=1}^{h} {\sum \nolimits }_{j=1}^{w} I_{gray}(i,j)$
*i*_*min*_	Minimum image intensity	*m* *i* *n*_1≤*i*≤*h*,1≤*j*≤*w*_ *I*_*gray*_(*i*,*j*)
*i*_*max*_	Maximum image intensity	*m* *a* *x*_1≤*i*≤*h*,1≤*j*≤*w*_ *I*_*gray*_(*i*,*j*)
*σ*	Standard deviation of intensity	$\sigma = \sqrt {\frac {1}{h*w} {\sum \nolimits }_{i=1}^{h} {\sum \nolimits }_{j=1}^{w} (i_{\mu } - I_{gray}(i,j))^{2}}$
*τ*_*o*_	Otsu’s threshold intensity	[29]
*e*_*o*_	Threshold effectiveness metric	[43]

## Endnotes

^1^ http://hamptonresearch.com

^2^ https://code.google.com/p/randomforest-matlab/

^3^ In the table, in order to calculate the number of experiments for a task, we used the notation: *η*_*n*_∗*η*_*c*_∗*η*_*f*_∗*η*_*r*_. In this notation, *η*_*n*_ refers to the number of normalizations that are applied to feature set, *η*_*c*_ refers to the number of classifiers used, *η*_*f*_ refers to the number of feature sets that are used for the corresponding experiments, and *η*_*r*_ is the number of repetition of the experiments.

## Appendix : list of features

In this appendix, the features used in our experiments are listed. Table 18 Appendix provides the list of intensity features. Histogram features are listed in Table 19 Appendix.

The texture features are provided in Table 20 Appendix. Let *N*_*g*_ denote the number of distinct green levels in the quantized image; *p*(*i*,*j*) represent the (*i*,*j*)^*th*^ entry in the normalized GLCM, *p*_*x*_(*k*) denote the *k* ^*th*^ entry of the matrix obtained by summing rows of *p*(*i*,*j*), and *p*_*y*_(*k*) represent the *k* ^*th*^ entry of the matrix obtained by summing columns of *p*(*i*,*j*). The following notation is used in the formulation of the features provided in Table 20.

**Table 19:** List of histogram features

Symbol	Description	Formulation
*μ*	Average image intensity	$\frac {1}{w*h}{\sum \nolimits }_{k=0}^{k=255}{k * H[k]}$
*σ*	Std devn of intensity	$\sqrt {\frac {1}{w*h}{\sum \nolimits }_{k=0}^{k=255}{ (k - \mu)^ 2 * H[k] }}$
*s*	Skewness	$\frac {1}{(w*h)*\sigma ^{1.0}} {\sum \nolimits }_{k=0}^{k=255}{ (k - \mu)^ 3 * H[k]}$
*k*	Kurtosis	$\frac {1}{(w*h)*\sigma ^{2}} {\sum \nolimits }_{k=0}^{k=255}{ (k - \mu)^ 4 * H[k] }$
v*E*	Entropy	-${\sum \nolimits }_{k=0}^{255} N[k] log(N[k]) \text {, where} N[k] = H[k]/(w*h)$
$g_{1}^{1}$, $g_{1}^{2}$, $g_{1}^{3}$,.. $g_{3}^{3}$	GLCM auto-correlation	Eq. 5
*i* *a*_1_, *i* *a*_2_, *i* *a*_3_	Image auto-correlation	Eq. 6
*m* *g*_1_, *m* *g*_2_, *m* *g*_3_	GLCM power spectrum magnitude	*m* *g*_*i*_=*m* *e* *a* *n*2(|*f* *f* *t* *s* *h* *i* *f* *t*(*f* *f* *t*2(*P*_*i*_))|),1≤*i*≤3
*mi*	Image power spectrum magnitude	*m* *i*=*m* *e* *a* *n*2(|*f* *f* *t* *s* *h* *i* *f* *t*(*f* *f* *t*2(*I*))|.^2^)

**Table 20:** List of texture features

	Feature	Formulation
*f*_1_	Autocorrelation [40]	$ {\sum \nolimits }_{i}{\sum \nolimits }_{j}(ij)p(i,j)$
*f*_2_	Contrast [40]	${\sum \nolimits }_{n=0}^{N_{g}-1}n^{2}\left \{ {\sum \nolimits }_{i=1}^{N_{g}} {\sum \nolimits }_{j=1}^{N_{g}}p(i,j) \left.\right | |i-j|=n\right \}$
*f*_3_	Correlation (Matlab) [43]	${\sum \nolimits }_{i}{\sum \nolimits }_{j} \frac {(i-\mu _{x})(j-\mu _{y})p(i,j)}{\sigma _{x} \sigma _{y}}$
*f*_4_	Correlation [40]	${\sum \nolimits }_{i}{\sum \nolimits }_{j} \frac {(ij)p(i,j)-\mu _{x}\mu _{y}}{\sigma _{x}\sigma _{y}}$
*f*_5_	Cluster prominence [41]	${\sum \nolimits }_{i}{\sum \nolimits }_{j}\left (i + j - \mu _{x} - \mu _{y} \right)^{4} p\left (i,j \right)$
*f*_6_	Cluster shade [41]	${\sum \nolimits }_{i}{\sum \nolimits }_{j}\left (i + j - \mu _{x} - \mu _{y} \right)^{3} p\left (i,j \right)$
*f*_7_	Dissimilarity [41]	${\sum \nolimits }_{i}{\sum \nolimits }_{j}\left | i-j \right |\cdot p(i,j)$
*f*_8_	Energy [40]	${\sum \nolimits }_{i}{\sum \nolimits }_{j}p(i,j)^{2}$
*f*_9_	Entropy [41]	$-{\sum \nolimits }_{i}{\sum \nolimits }_{j}p\left (i,j \right)\log \left (p(i,j)\right)$
*f*_10_	Homogeneity (Matlab) [43]	${\sum \nolimits }_{i}{\sum \nolimits }_{j} \frac {p(i,j)}{1+\left | i-j \right |}$
*f*_11_	Homogeneity [41]	${\sum \nolimits }_{i}{\sum \nolimits }_{j}\frac {1}{1+\left (i-j \right)^{2}}p\left (i,j \right)$
*f*_12_	Maximum probability [41]	$\underset {i,j}{MAX}p\left (i,j \right)$
*f*_13_	Sum of squares: Variance [40]	${\sum \nolimits }_{i}{\sum \nolimits }_{j}(i-\mu)^{2} p(i,j)$
*f*_14_	Sum average [40]	${\sum \nolimits }_{i=2}^{2N_{g}}i p_{x+y}(i)$
*f*_15_	Sum entropy [40]	$-{\sum \nolimits }_{i=2}^{2N_{g}}p_{x+y}(i)\log \left \{ p_{x+y}(i) \right \}$
*f*_16_	Sum variance [40]	${\sum \nolimits }_{i=2}^{2N_{g}}(i-f_{15})^{2}p_{x+y}(i)$
*f*_17_	Difference variance [40]	*v* *a* *r*(*p*_*x*−*y*_)
*f*_18_	Difference entropy [40]	$-{\sum \nolimits }_{i=0}^{N_{g}-1}p_{x-y}(i)\log \left \{ p_{x-y}(i) \right \}$
*f*_19_	Information measure of correlation 1 [40]	$\frac {HXY-HXY1}{max\left \{ HX,HY \right \}}$
*f*_20_	Information measure of correlation 2 [40]	(1−exp[−2(*H* *X* *Y*2−*H* *X* *Y*)])^1/2^
*f*_21_	Inverse difference (INV) [42]	${\sum \nolimits }_{i}{\sum \nolimits }_{j} \frac {p(i,j)}{1+\left | i-j \right |}$
*f*_22_	Inverse difference normalized [42]	${\sum \nolimits }_{i}{\sum \nolimits }_{j}\frac {p(i,j)}{1+\left | i-j \right |/N_{g}}$
*f*_23_	Inverse difference moment [42]	${\sum \nolimits }_{i}{\sum \nolimits }_{j} \frac {p(i,j)}{1+((i-j)/N_{g})^{2}}$

**Table 21:** List of global binary image features

Symbol	Description	Formulation
*N*_*f*_	No of white pixels in *B*	${\sum \nolimits }_{x=1}^{h}{\sum \nolimits }_{y=1}^{w}B(x,y)$
*μ*_*f*_	Foreground avg intensity	$\frac {1}{N_{f}} {\sum \nolimits }_{i=1}^{h} {\sum \nolimits }_{j=1}^{w} I_{gray}(i,j).B(i,j)$
*σ*_*f*_	Foreground std devn intensity	$\sqrt {\frac {1}{N_{f}} {\sum \nolimits }_{i=1}^{h}{\sum \nolimits }_{j=1}^{w} ((\mu _{f} -I_{gray}(i,j)).B(i,j))^{2}}$
*μ*_*b*_	Background avg intensity	$\frac {1}{h*w-N_{f}} {\sum \nolimits }_{i=1}^{h} {\sum \nolimits }_{j=1}^{w} I_{gray}(i,j)(1-B(i,j))$
*σ*_*b*_	Background std devn intensity	$\sqrt {\frac {1}{h*w-N_{f}} {\sum \nolimits }_{i=1}^{h} {\sum \nolimits }_{j=1,B(i,j)=0}^{w} ((\mu _{b} - I_{gray}(i,j)).(1-B(i,j))^{2}}$
*N*	Number of blobs	No. of connected components
*r*_*c*_	Image fullness	*c* *o* *n* *v* *e* *x* *H* *u* *l* *l* *A* *r* *e* *a*(*B*)/(*h*∗*w*)

**Table 22:** List of blob features

Symbol	Description	Formulation
$\mu _{o}^{i}$	Average intensity of *O*_*i*_	$\frac {1}{w_{i} \times h_{i}} {\sum \nolimits }_{j=m^{i}_{x} - w^{i}/2}^{m^{i}_{x} + w^{i}/2} {\sum \nolimits }_{k=m^{i}_{y} - h^{i}/2}^{m^{i}_{y} + h^{i}/2} I_{gray}(j,k)$
$\sigma _{o}^{i}$	Std devn of intensity of *O*_*i*_	$\sqrt {\frac {1}{w_{i} \times h_{i}} {\sum \nolimits }_{j=m^{i}_{x} - w^{i}/2}^{m^{i}_{x} + w^{i}/2} {\sum \nolimits }_{k=m^{i}_{y} - h^{i}/2}^{m^{i}_{y} + h^{i}/2} \left (\mu _{o}^{i} - I_{gray}(j,k)\right)^{2} }$
$N_{o}^{i}$	No of pixels in *O*_*i*_	*h*_*i*_ * *w*_*i*_
$Nf_{o}^{i}$	No of white pixels in *O*_*i*_	${\sum \nolimits }_{x=1}^{h_{i}}{\sum \nolimits }_{y=1}^{w_{i}}O_{i}(x,y)$
$p_{o}^{i}$	Perimeter of *O*_*i*_	${\sum \nolimits }_{i=1}^{h_{i}}{\sum \nolimits }_{j=1}^{w_{i}}\Omega _{i}(x,y)$
$ch_{o}^{i}$	Convex hull area of *O*_*i*_	[43]
$e_{o}^{i}$	Blob eccentricity of *O*_*i*_	[43]
$be_{o}^{i}$	Blob extent of *O*_*i*_	[43]
$bd_{o}^{i}$	Equivalent circular diameter of *O*_*i*_	[43]

**Table 23:** Graph features

Feature	Symbol	Description	Formulation
*Edge [44]	*η*	No of graphs (connected edges)	*η*=|*S*|
	*η*_1_	No of graphs with a single edge	*η*_1_=|*S*_*i*_|, where |*L*(*S*_*i*_)|=1
	*η*_2_	No of graphs with 2 edges	*η*_2_=|*S*_*i*_|, where |*L*(*S*_*i*_)|=2
	*η*_*c*_	No of graphs whose edges form a cycle	*η*_*c*_=|*S*_*i*_|,where *S*_*i*_ is a cyclic graph
	*η*_*p*_	No of line normals	$\eta _{p}=\sum \! \perp \! \left (S_{k} \right),\! \perp \! \left (S_{k} \right)\! =\! \left \{\!\! \begin {array}{lc} 1 & \exists l_{i}\in L_{k}\text {and }\exists l_{j}\in L_{k}\text {and} \\ & 70\leq \alpha \left (l_{i},l_{j} \right)\leq 90 \\ 0&otherwise \end {array}\right.$
	*μ*_*l*_	Average length of edges in all segments	$\mu _{l}=\frac {{\sum \nolimits }_{i\in \textbf {L}}l_{i}}{\left |\textbf {L}\right |}$
	*S*_*l*_	Sum of lengths of all edges	$S_{l}={\sum \nolimits }_{i\in \textbf {L}}l_{i}$
	*l*_*max*_	Maximum length of an edge	*l*_*max*_= max1≤*i*≤|**L**|(*l*_*i*_)
	*c*_*o*_	1 if *η*_*c*_>0, 0 otherwise	*c*_*o*_=∃*S*,*S*is a cyclic graph
	*l*_*o*_	1 if *η*_*p*_>0, 0 otherwise	*l*_*o*_=(∃*l*_*i*_∈*L*_*k*_and ∃*l*_*j*_∈*L*_*k*_and70≤*α*(*l*_*i*_,*l*_*j*_)≤90)
	*η*_*hc*_	No of Harris corners	[48]
*Hough	*η*_*hl*_	No of Hough lines	[49]
	*μ*_*hl*_	Average length of Hough lines	[49]

- $p_{x+y}(k) = {\sum \nolimits }_{i=1}^{N_{g}} {\sum \nolimits }_{j=1}^{N_{g}}p(i,j) \left | i+j = k \right.$
- $p_{x-y}(k) = {\sum \nolimits }_{i=1}^{N_{g}} {\sum \nolimits }_{j=1}^{N_{g}}p(i,j) \left | |i - j| = k\right.$
- $\mu _{x} = {\sum \nolimits }_{i}{\sum \nolimits }_{j}i\cdot p(i,j)$
- $\mu _{y} = {\sum \nolimits }_{i}{\sum \nolimits }_{j}j\cdot p(i,j)$
- $\sigma _{x} = {\sum \nolimits }_{i}{\sum \nolimits }_{j}(i-\mu _{x})^{2} \cdot p(i,j)$
- $\sigma _{y} = {\sum \nolimits }_{i}{\sum \nolimits }_{j}(j-\mu _{y})^{2} \cdot p(i,j)$
- $HXY = -{\sum \nolimits }_{i}{\sum \nolimits }_{j}p\left (i,j \right)\log \left (p\left (i,j \right)\right)$
- *HX* and *HY* are entropies of *p*_*x*_ and *p*_*y*_
- $HXY1 = -{\sum \nolimits }_{i}{\sum \nolimits }_{j}p\left (i,j \right)\log \left \{ p_{x}(i) p_{y}(i) \right \}$
- $HXY2 = -{\sum \nolimits }_{i}{\sum \nolimits }_{j}p_{x}(i) p_{y}(i)\log \left \{ p_{x}(i) p_{y}(i) \right \}$

In Table 20, the Matlab homogeneity feature (*f*_10_) and inverse difference feature (*f*_21_) are actually two different labels and implementations of the same feature. Although both features were extracted for our experiments, one of these features can be eliminated based on the programming environment.

**Table 24:** Shape-adaptive DCT features

Symbol	Description
$C_{m}^{i}$	Maximum of non-zero coefficients of SA-DCT of *O*_*i*_
$C_{\mu }^{i}$	Average of non-zero coefficients of SA-DCT of *O*_*i*_
$C_{N}^{i}$	No. of non-zero coefficients of SA-DCT of *O*_*i*_

The global region features and blob features are provided in Tables 21 and 22 Appendix, respectively. Table 23 Appendix provides graph related features, where *S* is the set of graphs in *I*, *S*_*i*_ the *i* ^*th*^ graph in *S*, **L** is the set of edges in *I*, |*L*(*S*_*i*_)| is the number of edges in graph *S*_*i*_, and *α*(*l*_*i*_,*l*_*j*_) represents the angle between *l*_*i*_ and *l*_*j*_. The list of shape adaptive features is provided in Table 24 Appendix.

## Abbreviations

BYS: Naïve Bayesian
CNN: Convolutional Neural Network
DCT: Discrete Cosine Transform
DFT: Discrete Fourier Transform
DT: Decision Tree Classifier
MBR: Minimum Bounding Rectangle
NN: Neural Network Classifier
Pacc: Probabilistic Accuracy
PCA: Principle Components Analysis
RF: Random Forest
SA-DCT: Shape-adaptive Discrete Cosine Transform
SVM: Support Vector Machine
